# Importance of Army and Navy Dentists

**Published:** 1859-07

**Authors:** 


### EDITORIAL DEPARTMENT.

*Importance of Army and Navy Dentists.*—At the last meeting of the American Dental Convention, a committee was appointed to memorialize Congress on the necessity of appointing dentists for the service of the army and navy. The resolutions authorising the appointment of this committee were offered by Dr. McKillops. We are glad that this subject has attracted the attention of the Convention, although to our certain knowledge the importance of it has been advocated by Dr. Maynard for these fifteen years past, at Washington City, and had so far impressed Mr. Fillmore in its favor that he brought it before the cabinet in council. The Secretaries of War and the Navy took some action in the matter. Nothing resulted from the former, and the latter went to work so stupidly and vexatiously as to make it necessary to countermand his orders in the matter as soon as his term of office expired, which happily occurred within a few days, and before his new "regulation" was issued. More recently, Mr. Davis, when Secretary of War, received the proposition as one of great value; and Mr. Dobbin, Secretary of the Navy, to whom he addressed a note upon the subject, at once, and heartily approved the proposition and expressed himself as under deep obligations to Dr. M. for bringing to his notice a project which his own sufferings from his teeth and benefit from their proper surgical treatment, convinced him was a most humane suggestion.

No official action however was taken. The time was not favorable. There had been pending before congress for some time, a bill affecting the corps of surgeons of the army, and it was thought best not to propose any thing that might defeat that bill. One of the army surgeons who favored the new proposition, visited the Surgeon General with Dr. M., and so far was he successful in advocating the project, that the

Surgeon General was willing to advocate the establishment of a corps of army dental surgeons of six to begin with, to be entirely distinct from the corps of surgeons in their duties, examinations, promotions and rank.

Although no official action has ever yet been had in the matter, the project is daily ripening. We have reason to know that Dr. M. misses no proper occasion to advocate and illustrate its propriety to such of his patients, from the President to the common soldier, as may have authority of place, official or legislative, or may be in any way affected by it. All approve the measure; and when the most favorable time arrives for legislative action, we have no doubt that Dr. M. will carry it through successfully.

